# Semi-Transparent Organic Photovoltaic Cells with Dielectric/Metal/Dielectric Top Electrode: Influence of the Metal on Their Performances

**DOI:** 10.3390/nano11020393

**Published:** 2021-02-04

**Authors:** Linda Cattin, Guy Louarn, Mustapha Morsli, Jean Christian Bernède

**Affiliations:** 1 Institut des Matériaux Jean Rouxel (IMN), Université de Nantes, CNRS, UMR 6502, 2 rue de la Houssinière, BP 92208, F-44322 Nantes, France; guy.louarn@cnrs-imn.fr; 2 Faculté des Sciences et des Techniques, Université de Nantes, 2 rue de la Houssinière, BP 92208, F-44000 Nantes, France; Saber.Morsli@univ-nantes.fr; 3 MOLTECH-Anjou, CNRS, UMR 6200, Université de Nantes, 2 rue de la Houssinière, BP 92208, F-44322 Nantes, France

**Keywords:** semi-transparent organic photovoltaic cells, dielectric/metal/dielectric transparent electrode, top transparent anode, MoO_3_/Ag/MoO_3_, inverted planar heterojunction, SubPc/C60 active layers

## Abstract

In order to grow semi-transparent organic photovoltaic cells (OPVs), multilayer dielectric/metal/dielectric (D/M/D) structures are used as a transparent top electrode in inverted OPVs. Two different electrodes are probed, MoO_3_/Ag/MoO_3_ and MoO_3_/Ag/Cu:Ag/ZnS. Both of them exhibit high transmission in visible and small sheet resistance. Semi-transparent inverted OPVs using these electrodes as the top anode are probed. The active organic layers consist in the SubPc/C_60_ couple. The dependence of the OPV performances on the top electrode was investigated. The results show that far better results are achieved when the top anode MoO_3_/Ag/MoO_3_ is used. The OPV efficiency obtained was only 20% smaller in comparison with the opaque OPV, but with a transparency of nearly 50% in a broad range of the visible light (400–600 nm). In the case of MoO_3_/Ag/Cu:Ag/ZnS top anode, the small efficiency obtained is due to the presence of some Cu diffusion in the MoO_3_ layer, which degrades the contact anode/organic material.

## 1. Introduction

The growing interest in organic photovoltaic cells (OPVs) is due to the fact that they possess some specific advantages such as light weight, intrinsic flexibility, and possible semi-transparency of organic thin films [1]. More specifically, semi-transparent OPVs attract strong interest due to the efforts currently directed toward building integrated photovoltaics (BIPVs). Recently, much research has focused on improving OPV efficiency [1,2,3,4], but some applications, such as BIPV, induce the use of semi-transparent OPVs, which are less efficient than the usual opaque OPVs because they have to transmit a significant amount of visible light [5,6]. Therefore, for semi-transparent OPVs, it is not appropriate to use optimal organic layer thickness. It is known that, the diffusion length of excitons being smaller than the organic layer thickness necessary to absorb light, the geometry of bulk heterojunction (BHJ) was used with success [7,8,9,10,11], while in the case of planar heterojunctions (PHJs), the excitons created too far from the electron donor/electron acceptor interface are lost, which, contrary to the case of BHJs, limits the thickness of PHJs and, therefore, their efficiency [12]. However, as said above, in the case of semi-transparent OPVs, we must not use the optimum thickness to preserve some OPV transparency, so we chose the PHJ–OPV configuration to grow semi-transparent devices. Such configuration allows for using small molecules and deposition under vacuum. Small molecules have a number of benefits such as easy preparation and purification and a unique final molecule that prevents the problem of batch-to-batch reproducibility [13,14,15]. Furthermore, the thermal deposition technique allows for easy fabrication of multilayer devices with precise control of the thickness of each layer [16,17]. Usually, the electrodes of opaque OPVs consist of a transparent conductive electrode (TCE), often an ITO (Indium Tin Oxide) thin film deposited onto a glass substrate, and a metal electrode, often aluminum. The high reflectivity of Al allows light to be reinserted into the organic layers. To achieve semi-transparent OPVs with acceptable performances, the main challenge is the substitution of a transparent electrode with high conductivity with a metal electrode [18]. Different solutions are possible, among them, dielectric/metal/dielectric (DMD) structures [19,20]. They possess quite equilibrated optical and electrical properties; moreover, they are easy to realize given the technique we have chosen to use via successive thermal sublimation/evaporation. In DMD, M allows obtaining small sheet resistance, but its high reflectivity strongly penalizes the light transmission. Thus, to obtain the transmission of the visible light, it is necessary to sandwich the M layer between two high refractive index dielectrics. MoO_3_ is one of the possible dielectrics; it is well known that it is very efficient as a hole-transporting layer (HTL) at the anode/electron donor interface [21,22]. Therefore, in the present work, MoO_3_ was chosen as the dielectric. As for the metal, Ag is commonly selected because it exhibits the highest metal conductivity coupled with the lowest absorption in the visible range among the metals [23]. Nevertheless, Ag is quite expensive, and it would be of high interest to use a cheaper metal. Therefore, we also used Cu, whose optical and electrical properties are very close to those of Ag. Unfortunately, Cu tends to diffuse into transition metal oxides such as MoO_3_ [24], so we tried to improve DCuD stability through the use of ZnS dielectric and Cu:Ag alloy [25]. After optimization, these DMD electrodes are used as top electrodes in inverted OPVs, such as ITO/Alq_3_/C_60_/SubPc/DMD. We chose the inverted OPV configuration due to its better stability [26]. The study shows that, even after the DMD structure optimization, the best results are obtained with DagD top electrodes. In comparison with the reference OPV, i.e., with Al as the top electrode, the efficiencies of the semi-transparent OPVs are only 20% smaller, but with a transparency of nearly 50% in a broad range of the visible light (400–600 nm).

## 2. Materials and Methods

The deposition and characterization techniques have already been described, and they are recalled in Supporting Information S1 and S2.

**Figure 1:**
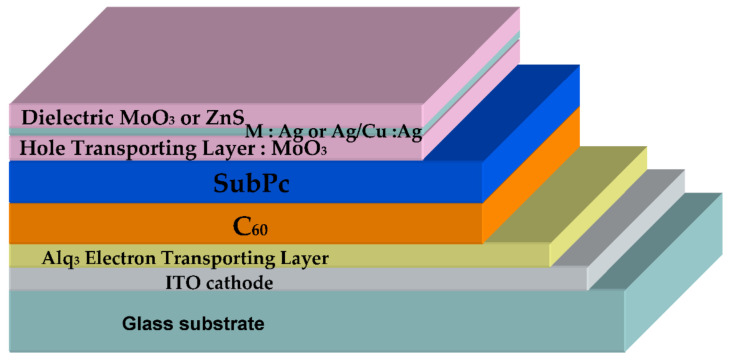
Typical inverted semi–transparent organic photovoltaic cell (OPV).

The inverted OPVs were deposited under vacuum (see Supporting Information S1), and they were as follows: ITO/Alq_3_/C_60_/SubPc/DMD (Figure 1). They are based on the electron acceptor/electron donor couple (EA/ED): C_60_/SubPc. SubPc is known for its high absorption coefficient and C_60_ for its high efficiency as an electron acceptor [27]. The thickness of these layers in inverted OPVs was earlier optimized: 40 nm for C_60_ and 16 nm for SubPc [28]. The buffer layer inserted between the cathode and the electron acceptor, which is called the exciton blocking layer (EBL) in the case of PHJ-OPVs [29], is a thin layer (9 nm) of Alq_3_. Often, the EBL consists in a bathocuproine layer [26]; however, it was shown that Alq_3_ allows obtaining more stable OPVs, which justifies our choice [30]. Regarding the DMD top electrode, the first dielectric layer also serves as a hole-transporting layer (HTL), so, as evocated above, it consists in a MoO_3_ layer. For the other layers of the MoO_3_/M/D structure, we used two metals: either Ag or Cu:Ag alloy and ZnS as the dielectric. The atomic concentration of Ag in the alloy was 5 at%. In the case of the reference opaque electrode, the top electrode was MoO_3_/Al. We have already optimized the DMD structures, either as transparent conductive structures or as bottom electrodes in opaque OPVs [23,25]. Here, due to the fact that the DMD structures were deposited onto stacked ITO/Alq_3_/C_60_/SubPc layers, we had to check the optimum thickness of the MoO_3_ interfacial layer.

The different devices were characterized using the following techniques: optical transmission and absorption measurements, electrical conductivity measurements, X-ray photoelectron spectroscopy, J-V characteristics of OPV in the dark, and under AM1.5 solar spectrum.

These different techniques are described in Supporting Information S2.

## 3. Results and Discussion

**Figure 2:**
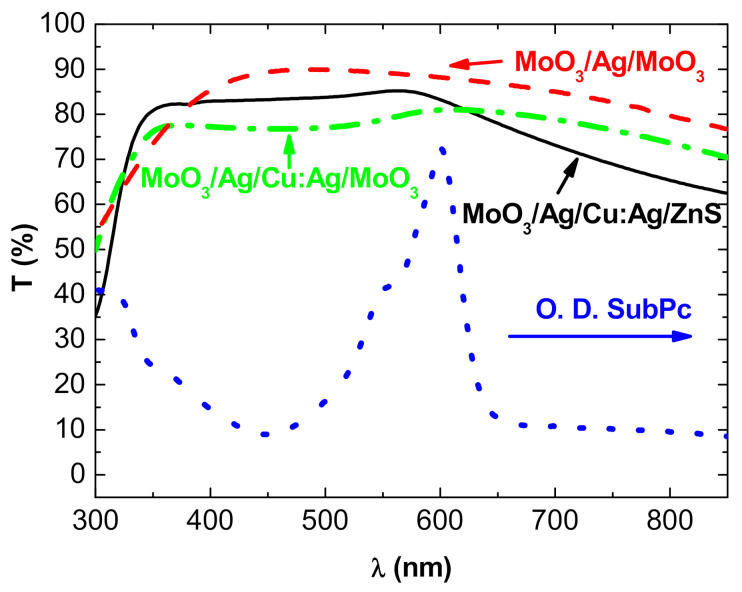
Transmission spectra of (**----**) glass/MoO_3_ (20 nm)/Ag (10 nm)/MoO_3_ (35 nm), (----) MoO_3_ (20 nm)/Ag (2 nm)/Cu:Ag (15 nm)/ZnS (45 nm) structures, and (**ꟷ•ꟷ•ꟷ**) MoO_3_ (20 nm)/Ag (2 nm)/Cu:Ag (15 nm)/MoO_3_ (45 nm) structures, and (•••) absorption spectrum of SubPc.

Regarding the DMD structures, when Ag was used as metal and MoO_3_ as dielectric, reproducible results were obtained. The performances of the structures Glass/MoO_3_ (20 nm)/Ag(10 nm)/MoO_3_ (35 nm) are summarized in Table 1, and the visible optical transmission is shown in Figure 2. 

From optical and electrical measurements, we have calculated Φ_M_, the figure of merit proposed by Haack using the empiric formulae [31]:

Φ_M_ = T^10^/σ_sh_ (1)

where Φ_M_ is the figure of merit, T is the transmission of light, and σ_sh_ is the sheet resistance.

It allows for comparing the “opto-electrical” performances of the different electrodes.

In the case of MoO_3_/Ag/MoO_3_ structures, the optimum thickness of the layers has been determined in previous publications to be 20 nm/10 nm/35 nm, respectively [23]. The thickness of the Ag layer, 10 nm, corresponds to the percolation threshold of the metal layer; for less thickness, the metal film is discontinuous; for thicker films, the reflectivity and absorption of the metal film increase. 

In the case of copper, it is not so easy to grow performing TCE. In fact, we have already shown that Cu diffuses easily and spontaneously into MoO_3_ [24]; therefore, in the structures MoO_3_/Cu/MoO_3_, we inserted ZnS in the MoO_3_ top layer in such a way that we used MoO_3_/Cu/ZnS structures, the diffusion of Cu in ZnS being far smaller than in MoO_3_. However, we cannot substitute ZnS for the other MoO_3_ layer because, while MoO_3_ is an excellent hole-extracting layer, ZnS is not, and the performances of the OPVs using ZnS/M/ZnS top electrodes were very poor. Thus, it is necessary to keep MoO_3_ as an interface layer between the SubPc layer and the metal layer. Nevertheless, since we know that Cu diffuses spontaneously into MoO_3_ [24], we introduced an ultra-thin Ag layer at the interface MoO_3_/Cu. We have already shown that such an ultra-thin Ag layer significantly improves the stability of MoO_3_/Cu structures [23]. Nevertheless, it is not sufficient to obtain really stable electrodes. Therefore, we used a Cu:Ag alloy. We have shown that the structures using such an alloy with 5 at.% of Ag exhibit acceptable stability [25]. Using dielectric layer thicknesses determined previously, typical results are shown in Table 1 and Figure 2. When the metal is Cu, regardless of the dielectric used, the general shape of the transmission curve is similar but, due to higher Cu diffusion in MoO_3_, the curve is flatter when the two dielectric layers are MoO_3_. It must be noted that in the case of copper, it is necessary to use a thicker metal film to obtain MoO_3_/M/ZnS structure with acceptable sheet resistance, which has a thickness of 17 nm. The figure of merit of the structures using Cu:Ag metal layer is slightly better with 2 nm of Ag than a structure with 1 nm of Ag. Moreover, 2 nm of Ag permits obtaining structures more stable than those with 1 nm of Ag. As already mentioned regarding the properties of DMD structures, there is a critical thickness of the M layer, which corresponds to the percolation threshold of the metal layer; for less thickness, the metal film is discontinuous and the structure is insulating; for thicker films, the sheet resistance decreases slowly but the light transmission decreases. In fact, the layer thickness corresponding to the percolation threshold of the electrical properties is also of the optimum thickness for optical properties. Below this thickness, the metal film is discontinuous, which results in enhanced absorbance due to plasmonic resonances, involving a decreased transmittance. For thicknesses beyond the threshold value, the reflectivity and absorbance of the metal layer increase, inducing a decreased transmission of light [21,23,32].

In Figure 2, we introduce the absorption curve of SubPc. It can be seen that the maximum of absorption of SubPc corresponds to the transmission maximum of the DMD structures, which can be favorable for a compromise between the absorption and transmission of semi-transparent OPVs. On the other hand, it can be seen that, on the ultraviolet side of the spectrum, the transmission is limited due to the optical band gap of the dielectrics, while in the near-infrared domain, the transmission decreases due to the plasma effect resulting from the high concentration of mobile electrons in the IR region, which allows having conductive electrodes. 

Therefore, at the beginning of the study of the semi-transparent OPVs, we used MoO_3_ (20 nm)/Ag (10 nm)/MoO_3_ (35 nm) and MoO_3_ (20 nm)/Ag (2 nm)/Cu:Ag (15 nm)/ZnS (45 nm) top electrodes. Unfortunately, the efficiency of the OPVs was quite low, with high-series resistances. MoO_3_ being resistive—a disappointing result—was attributed to the thickness of the interfacial MoO_3_ layer, 20 nm. Therefore, it was necessary to decrease this thickness. The experimental study shows that a thickness of 10 nm allows obtaining optimum results. 

**Figure 3:**
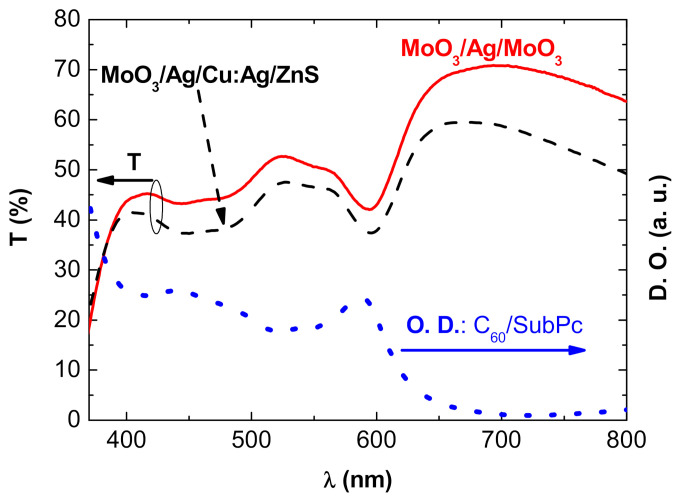
Transmission spectra of OPVs with different top electrodes: (**^____^**) MoO_3_ (10 nm)/Ag (10 nm)/MoO_3_ (35 nm) and (-----) MoO_3_ (10 nm)/Ag (2 nm)/Cu:Ag (15 nm)/ZnS (45 nm) and (**…..**) absorption spectrum of the OPV without top electrode: Glass/ITO/Alq_3_/C_60_/SubPc.

Figure 3 displays the spectral transmission of the complete OPVs: ITO/Alq_3_/C_60_/SubPc/DMD, the DMD top electrode being either MoO_3_ (10 nm)/Ag (10 nm)/MoO_3_ (35 nm) or MoO_3_ (10 nm)/Ag (2 nm)/Cu:Ag (15 nm)/ZnS (45 nm). 

Obviously, the transmission spectrum of the opaque OPV, with a 100 nm thick Al top electrode, is null. In the case of semi-transparent OPVs, i.e., when the top electrode is either MoO_3_ (10 nm)/Ag (10 nm)/MoO_3_ (35 nm) or MoO_3_ (10 nm)/Ag (2 nm)/Cu:Ag (15 nm)/ZnS (45 nm), the transmission is higher for wavelengths exceeding λ = 615 nm, due to the absorption spectrum shape of SubPc (Figure 3). After reaching a maximum value, the transmission gradually decreases, following the decreasing transmission curve of the DMD structures. On the whole spectrum, the transmission difference between the two types of OPVs follows the difference in transmission of the DMDs themselves. On the other hand, as expected, their minimum transmission, situated between 550 nm and 615 nm, corresponds to the domain of maximum absorption of SubPc. More globally, the transmission curve of the OPVs is the inverted image of the absorption curve, which confirms the relatively good transmission of light by the anodes used. 

**Figure 4:**
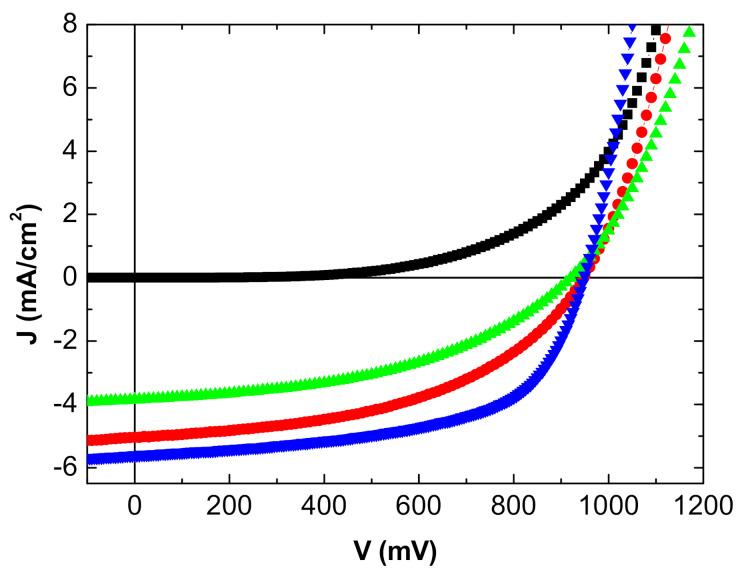
J-V characteristics inverted OPV with MoO_3_/Ag/MoO_3_ as top anode: in the dark (■), under light from the ITO side (●), and from the MoO_3_/Ag/MoO_3_ side (▲). Classical MoO_3_/Al top anode (▼).

**Figure 5:**
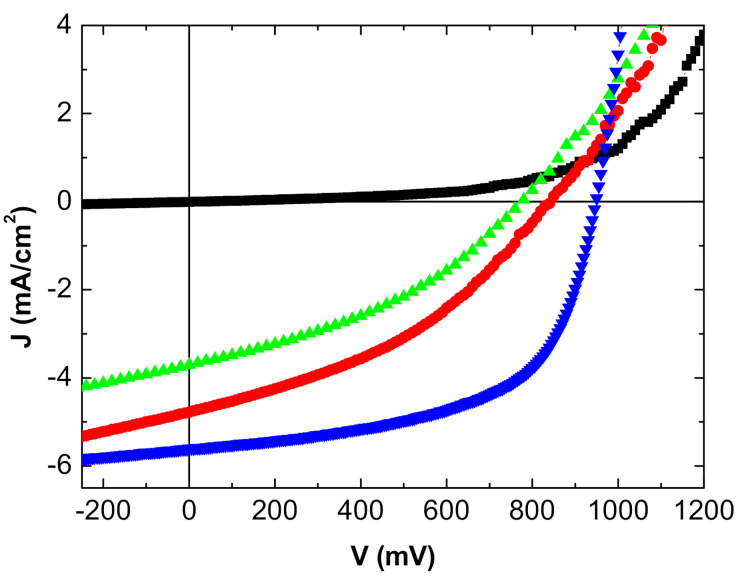
J-V characteristics inverted OPV with MoO_3_/Ag/Cu:Ag/ZnS as top anode: in the dark (■), under light from the ITO side (●) and from the MoO_3_/Ag/Cu:Ag/ZnS side (▲). Classical MoO_3_/Al top anode (▼).

The J-V characteristics of the inverted OPVs are shown in Figure 4 and Figure 5 and summarized in Table 2.

The reference OPV with the opaque Al top electrode gives the best results, which is not unexpected. Nevertheless, in the case of the MoO_3_/Ag/MoO_3_ top anode, when the OPV is illuminated on the ITO side, the efficiency reached is not too far from that obtained with the Al top electrode.

While the open circuit voltage Voc is stable, the efficiency decrease is due to the short circuit-current—Jsc—and the Fill Factor (FF). The reflectivity of the Al top anode is far higher than that of MoO_3_/Ag/MoO_3_, which justifies the decrease in Jsc.

Regarding FF, the Ag film thickness being only 10 nm, its homogeneity must be less than that of the 100 nm thick Al layer, which should induce interface traps and a higher sheet resistance as shown by the increase in series resistance. When deposited onto organic layers, the sheet resistance of the MoO_3_/Ag/MoO_3_ electrode is probably not as low as when deposited onto a polished glass substrate. 

This effect is reinforced when the OPV is illuminated on the MoO_3_/Ag/MoO_3_ side. To the effect of the low reflectivity of the electrode and higher sheet resistance, we must add the smaller light transmission; all these losses result in a deterioration of the OPV performances, mainly in a significant decrease in Jsc. 

In the case of MoO_3_/Ag/Cu:Ag/ZnS as the top anode, the performances obtained are far smaller than those of the reference OPV. In order to understand such poor results, we have proceeded to do some more characterization. We have already shown that the presence of Cu at the organic material/anode interface induces severe degradation of the OPVs, so we have studied the profile of the MoO_3_/Ag/Cu:Ag/ZnS structure. In order to reproduce the experimental conditions of the OPVs, we deposited onto a glass substrate the layer sequence MoO_3_/Ag/Cu:Ag/ZnS. Generally, when thin layers are superimposed, the higher the number of layers superimposed, the rougher the sample surface is. This makes it more difficult to interpret an XPS profile because following this roughness, we lose resolution. Subsequently, we chose to make the profile on a sample deposited on glass. 

**Figure 6:**
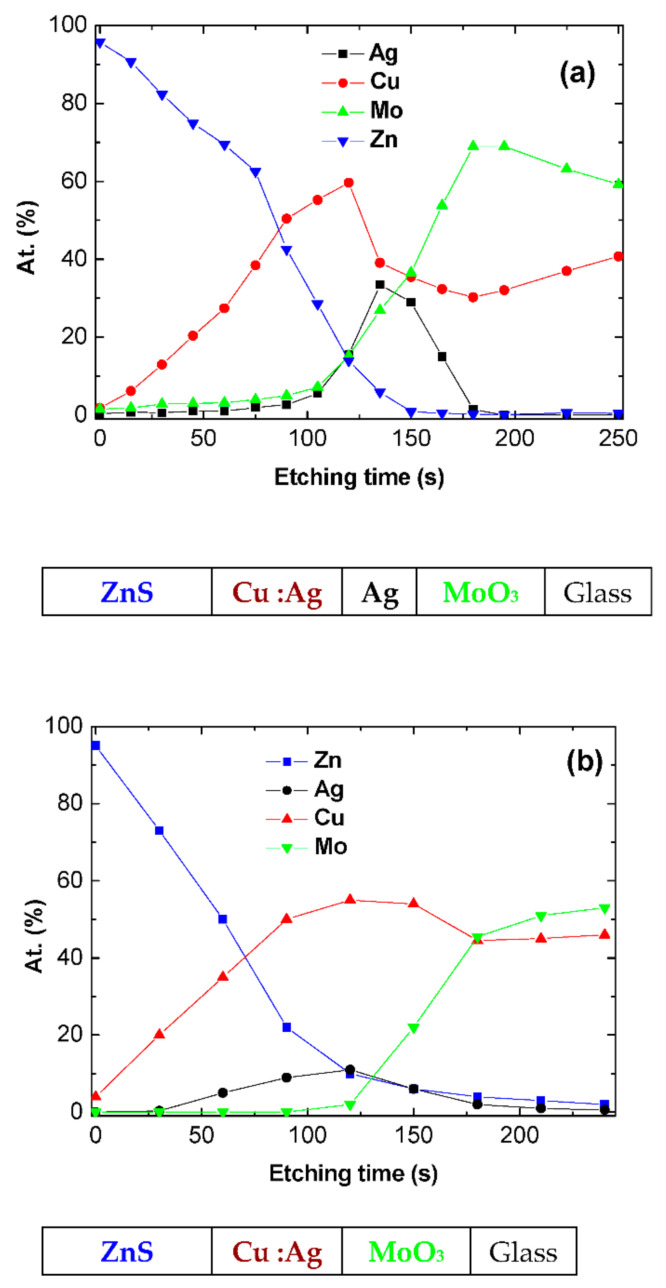
XPS profile of the electrodes MoO_3_/Ag/Cu:Ag/ ZnS (**a**) and MoO_3_/Cu:Ag/ZnS (**b**).

The XPS profile obtained on such glass/MoO_3_/Ag/Cu:Ag/ZnS multilayer structure is reported in Figure 6. To check the influence of the thin Ag layer introduced between the Cu and MoO_3_ layers, we also present the profile of a glass/MoO_3_/Cu:Ag/ZnS multilayer structure in Figure 6b. It can be seen that even if Ag limits the Cu diffusion into MoO_3_, without Ag, the profile Cu/Mo is flat, and there is a significant diffusion of Cu into Mo, with 40 at.% of Cu present at the interfacing electrode/organic material. Nevertheless, in comparison with Figure 6b, at the center of the structure, when the thin layer of Ag is present, the atomic concentration of Cu is higher, while, on the other hand, it is lower in the MoO_3_ layer. This shows the effectiveness of this thin Ag layer in limiting the diffusion of Cu. However, the presence of a relatively high concentration of Cu at the interface has a negative effect on the OPV performances. We have already shown that if an ultra-thin layer of 0.6 nm of Cu is an efficient anode buffer, this positive effect is destroyed when Cu is present in a thicker layer [33], which is the case at hand. The diffusion of Cu induces a decrease of the shunt resistance, resulting in poor rectifying properties. Moreover, the Cu diffusion results in an increase of the sheet resistance of the electrode. All that results in a decrease in the values of Voc, Jsc, FF, and efficiency. As in the case of MoO_3_/Ag/MoO_3_, the performances are smaller when the OPV is illuminated from the top anode side, for the same reasons.

The partial diffusion of Cu into MoO_3_ in the MoO_3_/Ag/Cu:Ag/ZnS structures justifies the fact that to obtain acceptable sheet resistance it is necessary to use thicker (17 nm) metal films in the case of these TCE, than in the case of MoO_3_/Ag/MoO_3_.

Nevertheless, it must be underlined that, as regards results obtained with the MoO_3_/Ag/MoO_3_ anodes, the results obtained are at the level of results already published [34,35,36,37], but using here the simple PHJ–OPV configuration and based on well-known and inexpensive molecules. 

## 4. Conclusions

It can be said that, up to now, if DMD structures using Ag as metal and MoO_3_ as dielectric have been demonstrated to be highly efficient transparent electrode on top of OPVs, the results obtained with MoO_3_/Ag/Cu:Ag/ZnS electrodes are not as convincing. Such a disappointing result is attributed to the fact that copper tends to diffuse in MoO_3_, even if the use of Cu:Ag alloy permits limiting the Cu diffusion. That said, the results obtained with MoO_3_/Ag/MoO_3_ top electrode are far most promising. An optimized MoO_3_/Ag/MoO_3_ top electrode allows achieving semi-transparent OPVs with quite high transparency. Of course, this transparency penalizes the short circuit current and, therefore, the OPV efficiency; nevertheless, the performances of these OPVs put them among the most transparent OPVs for a yield of the same order of magnitude as those made with cells based on BHJ. In fact, since, in order to obtain semi-transparent OPVs, it is necessary to limit the thickness of the organic materials, the present work shows that the benefit of using BHJ, which is very efficient in classical OPV, is lost here. In the case of semi-transparent OPVs, planar heterojunction appears to be a promising solution, paving the way toward building integrated photovoltaics (BIPVs) for their ease of production and high reproducibility.

In the case of MoO_3_/Ag/Cu:Ag/ZnS electrodes, the performances of the DMD are diminished due to partial Cu diffusion into the dielectrics, mainly into MoO_3_. In order to improve the power conversion of OPV using a top electrode containing Cu, we are now probing new DMD configurations:

- Since Cu diffusion in ZnS is far smaller than in MoO_3_, we are going to probe MoO_3_/ZnS/Ag/Cu:Ag/ZnS structures. The bilayer of dielectric MoO_3_/ZnS has a dual goal:
  - MoO_3_ allows an efficient collection of holes;
  - ZnS minimizes the diffusion of Cu.

This should improve the efficiency of the cells.

Another possibility is to use another Cu alloy; we aim to replace Cu:Ag with other alloys such as Cu:Cr, as Cr is well-known as a diffusion barrier.

## Figures and Tables

**Table 1:** Main parameters of the transparent electrodes.

Transparent Electrode	Sheet Resistance (Ω/sq)	Maximum Transmission (%)	Figure of Merit 10^−3^(Ω)^−1^
ITO	15	93.5	34
MoO_3_/Ag/MoO_3_	5	90.1	70
MoO_3_/Ag (1 nm)/Cu:Ag (16 nm)/ZnS	33	85.2	6.1
MoO_3_/Ag (2 nm)/Cu:Ag (15 nm)/ZnS	29	83.9	6.0

**Table 2:** Parameters of the inverted OPVs using different top anodes.

Anode	Light Side	Voc	Jsc	FF	η	Rs	Rsh
Al/MoO_3_	ITO	0.94	5.60	57	3.00	20	1800
MoO_3_/Ag/MoO_3_	ITO	0.94	5.04	48	2.34	30	1200
MoO_3_/Ag/MoO_3_	DMD	0.92	3.85	45	1.61	35	1000
MoO_3_/Ag/Cu:Ag/ZnS	ITO	0.84	4.77	40	1.55	40	700
MoO_3_/Ag/Cu:Ag/ZnS	DMD	0.43	3.71	39	1.10	40	500

## Data Availability

Data are available.

## Supplementary Materials

The following are available online at https://www.mdpi.com/2079-4991/11/2/393/s1, S1: Preparation of the substrates and deposition conditions; S2: Characterization techniques.
