# Supplementary material for: Semi-Transparent Organic Photovoltaic Cells with Dielectric/Metal/Dielectric Top Electrode: Influence of the Metal on Their Performances
Source: Nanomaterials (Basel). 2021 Feb 4;11(2):393. doi: 10.3390/nano11020393 (PMC7913718; doi:10.3390/nano11020393)
Supplement: Supplementary file 1 [file nanomaterials-11-00393-s001.pdf]

nanomaterials

MDPI

Supporting Information

Semi-Transparent Organic Photovoltaic Cells with Dielectric/Metal/Dielectric Top Electrode: Influence of the Metal on Their Performances

Linda Cattin [1,*], Guy Louarn [1], Mustapha Morsli [2] and Jean Christian Bernède [3,*]

[1] Institut des Matériaux Jean Rouxel (IMN), Université de Nantes, CNRS, UMR 6502, 2 rue de la Houssinière, BP 92208, F-44322 Nantes, France; guy.louarn@cnrs-imn.fr
[2] Faculté des Sciences et des Techniques, Université de Nantes, 2 rue de la Houssinière, BP 92208, F-44000 Nantes, France
[3] MOLTECH-Anjou, CNRS, UMR 6200, Université de Nantes, 2 rue de la Houssinière, BP 92208, F-44322 Nantes, France
* Correspondence: Linda.Cattin-Guenadez@univ-nantes.fr (L.C.); jean-christian.bernede@univ-nantes.fr (J.C.B.); Tel.: +33-51-12-55-31S.1 Preparation of the substrates and deposition conditions

S.1 Preparation of the substrates and deposition conditions

ITO coated glass and bare glass substrates, after scrubbing with soap, were rinsed in running deionised water. Then, they were dried with an argon flow, heated for 10 min at 100 °C, and finally loaded into a vacuum chamber (10^{-4} Pa). The size of the samples was 2.5 cm × 2.5 cm (or 1.6 cm). The substrates coated by ITO were provided by SOLEMS.

For DMD Structures, the layers were successively deposited, using an evaporation apparatus, into a vacuum chamber (10^{-4} Pa), onto substrates at room temperature, using tungsten crucibles, loaded with dielectric powders or metal wires. The MoO_3 bottom dielectric layer thickness was 20 nm, while the top dielectric layer was 35 nm thick in the case of MoO_3 or 45 nm in the case of ZnS. After deposition of the bottom layer, at a deposition rate of 0.06 nm/s, metal film was deposited by thermal evaporation at a deposition rate of 0.6 nm/s. Subsequently, the top dielectric layer was deposited at identical conditions to those used for the bottom layer. Both, film thickness and deposition rate, were monitored with a quartz crystal microbalance.

About the organic photovoltaic cells deposition process, all the layers were deposited in the same apparatus. Following earlier studies, the deposition rate and the thickness were 0.1 nm/s, 9 nm for Alq_3, 0.05 nm/s, 40 nm for C_{60} and 0.03 nm/s, 9 nm for SubPc After organic thin film deposition, the DMD top electrode was deposited as described above. It must be noted that the deposition apparatus is equipped with eight deposition sources and a mobile substrate holder, which allows depositing the whole OPV devices in the same run.

S.2 Characterization techniques

-Sheet resistance Rsh, of the D/M/D structures was measured at room temperature, using a four-point technique.

-The optical measurements were carried out at room temperature using a UV/visible spectrometer (PERKIN ELMER Lambda 1050 spectrophotometer). The optical transmission could be measured in 300 nm–2500 nm spectral range.

-Electrical characterizations of the OPVs were performed with an automated I-V tester, in the dark and under sun global AM 1.5 simulated solar illumination. Performances of photovoltaic cells were measured using a calibrated solar simulator (Oriel 300W) at 100 mW/cm² light intensity adjusted with a PV reference cell (0.5 cm² CIGS solar cell, calibrated at NREL, USA). Measurements were performed at an ambient atmosphere. All devices were illuminated through TCO electrodes.

The slopes of the electrical characteristics at the short circuit point and at the open circuit voltage are the inverse values of the shunt resistance (Rsh) and the series resistance (Rs) cell respectively.

- XPS measurements were carried out at room temperature on an Axis Nova spectrometer (Kratos Analytical) using the Al Ka line (1486.6 eV) as the excitation source. Survey spectra were acquired at pass energies of 80 eV (energy resolution of 0.87 eV). Data analysis was performed using CasaXPS software. Binding energy for the C 1s hydrocarbons peak was set at 285 eV in the calibration procedure. Then data were analysed with the CasaXPS software.

The depth profile of the structures was studied by recording successive XPS spectra obtained after argon ion etching for short periods. Sputtering was accomplished at pressures of less than2 × 10^{-6} Pa, 47 μA/cm2 current density and a 4 kV beam energy using an ion gun. With these experimental conditions, all the analysed surface was sputtered (raster size: 3 × 3 mm^2).

References

1. Z. ElJouad, L. Barkat, N. Stephant, L. Cattin, N.Hamzaoui, A. Khelil, M.Ghamnia, M. Addou, M. Morsli, S. Béchu, C. Cabanetos, M. Richard-Plouet, P. Blanchard, J.C. Bernède, Ca/Alq3 hybrid cathode buffer layer for the optimization of organic solar cells based on a planar heterojunction, *Journal of Physics and Chemistry of Solids*. **2016**, 98, 128–135
